# “So let me give you money, you give me what I want”: decision-making priorities around contraceptive method and source choice among young women in Kenya

**DOI:** 10.1186/s12978-023-01641-9

**Published:** 2023-06-26

**Authors:** Lisa M. Calhoun, Mahua Mandal, Bernard Onyango, Erick Waga, Courtney McGuire, Thomas van den Akker, Lenka Beňová, Thérèse Delvaux, Eliya M. Zulu, Ilene S. Speizer

**Affiliations:** 1 Carolina Population Center, University of North Carolina at Chapel Hill, 123 West Franklin Street, Suite 210, NC 27516 Chapel Hill, USA; 2 Athena Institute, Vrije Universiteit, Amsterdam, The Netherlands; 3 Department of Public Health, Institute of Tropical Medicine, Antwerp, Belgium; 4 African Institute for Development Policy, Nairobi, Kenya; 5 Department of Obstetrics and Gynecology, Leiden University Medical Center, Leiden, The Netherlands; 6 Department of Maternal and Child Health, Gillings School of Global Public Health, University of North Carolina at Chapel Hill, Chapel Hill, NC USA

**Keywords:** Family planning, Youth, Method choice, Method source, Service delivery point

## Abstract

**Background:**

Many factors influence young women’s choice of contraceptive methods and where to source them, yet less is known about whether one of these choices (method or source) is prioritized and the relationship between these choices. This study qualitatively explored decision-making around contraceptive method and source choice among young women in Kenya.

**Methods:**

In August–September 2019, 30 in-depth interviews were conducted with women ages 18–24 who had used two or more contraceptive methods and resided in three counties: Nairobi, Mombasa or Migori. Participants were recruited from public and private health facilities and pharmacies. Interview guides captured information about decision-making processes for each contraceptive method the respondent had ever used. Responses were audio-recorded, transcribed, translated into English, coded, and analyzed thematically.

**Results:**

The majority of respondents knew which method they wanted to use prior to seeking it from a source. This was true for all types of methods that women ever used. Of the small number of respondents who selected their source first, most were in the post-partum period or experiencing side effects and sought counseling at a source before choosing a method.

**Conclusions:**

This study highlights the importance of providing young women with high quality counseling that provides full information about contraceptive options and addresses that young women’s needs vary along the reproductive health continuum of care. This will ensure that young women have information to inform future contraceptive decision-making prior to seeking care.

## Background

In recent years, the global family planning (FP) community has galvanized around ensuring accessible, high quality FP care for adolescents and youth, including access to a full range of contraceptive methods [1, 2]. A well-established literature base shows that a range of factors influence contraceptive use among young women. These include demand-side factors related to their social environment [3–5]; knowledge and awareness of specific methods [6–9]; attitudes towards FP of the young person and her peers, partner or family [7, 9, 10]; and women’s own fertility and FP intentions [7, 10], amongst others. Yet, when young women make the decision to avoid a pregnancy, there are at least two critical choices that must be made: which contraceptive method to use and where to obtain it. In this paper, we are interested in exploring these two decisions—method choice and source choice—and if and when one decision takes priority and then subsequently influences the other decision.

Various features of sources or service delivery points (SDPs) have been identified as influential on where young women seek contraceptive methods. Some of these supply-side factors include concerns about privacy and confidentiality [9, 11, 12]; geographic accessibility of the SDP and the flexibility of its operating hours [7, 9, 13]; cost [9, 11]; method availability and stock-outs, which are closely tied to the type of facility selected [9, 14]; and quality of care and expectations for provider interactions [15–18]. Several of these factors may drive young people to seek FP services at a specific type of source that is more convenient, more discreet, or less costly (e.g., a pharmacy, drug shop, or a public sector facility), though less is known about young people’s decision-making around sourcing contraception from pharmacies. Young people’s choice of a method is then limited to the methods available at the selected site.

Radovich and colleagues analyzed Demographic and Health Surveys between 2000 and 2016 from 33 sub-Saharan African nations and noted the link between type of contraceptive method that young women used and source type; they found that 80% of male condom users reported procuring their method from a drug shop or informal provider, while young women who used IUDs and implants overwhelmingly sourced them from public health facilities [19]. While there appears to be clear patterns for obtaining certain types of methods from specific sources, there is more to be learned regarding the process for and influences on the sequencing of these decisions. This includes better understanding of whether method choice or source choice is prioritized; that is, whether young women first select the method they want and then decide where to obtain it or whether they initially determine where they will seek FP services and subsequently choose a method among those available at that site.

Research on how and if young women prioritize decision-making about method choice and source choice is limited. Prior studies from Nigeria and Ghana using exit interviews with FP clients of all ages found that the majority of women seeking a FP method for the first time had a preference for a particular method before coming to the SDP [20, 21]. However, these studies exclude users who went to pharmacies or shops, frequently for male condoms or emergency contraceptives (EC), and often do not examine whether women go to a specific SDP and choose among the methods available or if they know what method they want and choose the SDP accordingly. A study by Jarvis and colleagues in the Democratic Republic of Congo showed that a higher percentage of clients reported a pre-existing preference for implants and IUDs among those who sought care at outreach events or special FP days as compared to those who sought care at a facility; the authors suggest this may be due to clients knowing that these methods would be available at such events and therefore decided to attend [22]. These few studies focus on women of all ages, without a specific focus on young women under age 25, and do not attempt to determine how and when source factors into the choice of method.

The objectives of this study are to understand whether young women prioritize their choice of contraceptive method or method source and how one choice influences the other in the contraceptive decision-making process in Kenya. Given that the choice of method to use and where to source a method are not completely independent, we are interested in the intersection of these decisions and whether one of these decisions takes priority over the other for young women who want to delay or avoid pregnancy. Better understanding of this decision-making process among young women will help in the development of new program strategies, including those which focus on increasing knowledge about and access to a range of contraceptive methods among young people.

### Context

In Kenya, the site of this study, more than 60% of the population is under 24 years of age [23]. Over the last several years, adolescent pregnancy has been a pressing public concern in Kenya with increasing attention during the COVID-19 pandemic [24, 25]. According to the 2022 Kenya Demographic and Health Survey, 15% of young women ages 15–19 had ever been pregnant and an additional 3% were currently pregnant [26]. In 2020, 11.7% of female adolescents ages 15–19 and 45.8% of women ages 20–24 years reported current use of a modern contraceptive method [23]. Despite different levels of use, contraceptive method mix was similar among these two age groups with implants, injectables and male condoms being the most commonly used methods. Notably, method mix varies considerably by marital status with unmarried women ages 15–24 reporting highest use of male condoms (15.2%) and married women ages 15–24 reporting highest use of implants and injectables (25.5% and 23.4%, respectively) [23].

Kenya has a six-tier pyramidal health system with community facilities at the lowest level, followed by dispensaries, health centers, county hospitals, county referral hospitals, and national referral hospitals being the highest tier. The Government of Kenya provides all methods of contraception free of charge at public facilities, yet a study by Radovich et al. found that nearly half of modern contraceptive users paid user fees when seeking FP from a public facility [27]. More than 80% of public facilities in Kenya reported having oral contraceptives, injectables, male condoms, IUDs and implants available, yet 23% reported stock-outs of these commodities for an average of six days per month [28]. A policy change in 2018 permitted injectables to be provided pharmacies [29]. Approximately 60% of women under 25 years of age source their contraceptive method from the public sector and 40% from the private sector, which includes private facilities, and pharmacies (locally referred to as chemists) [30]. Pharmacies are common sources for male condoms, oral pills and emergency contraception, though these methods are also available from the public sector [23].

## Methods

### Study design

This paper uses data collected as part of a cross-sectional qualitative study for the Full Access, Full Choice project in Kenya, which was implemented by the Carolina Population Center at the University of North Carolina at Chapel Hill (UNC) and the African Institute for Development Policy (AFIDEP). The aim of the Full Access, Full Choice project is to generate evidence on expanded contraceptive method choice among young people.

The overall study aimed to understand influences on young women’s contraceptive use decisions and behaviors from the time they first used a contraceptive method until the time of interview. A detailed description of the Full Access, Full Choice (FAFC) project, the study design and additional findings from this study can be found elsewhere [31]. The FAFC project team was comprised of American and Kenyan researchers, all of whom were knowledgeable and experienced in sexual and reproductive health research in Kenya; differing perspectives of the study team may have affected our overall ability to objectively design, analyze and report study findings. Power differentials and dynamics within the study team were discussed and addressed throughout the course of the project, with emphasis on understanding and valuing individual types of knowledge and experience that team members brought. The overall study had an external advisory group that employed a transdisciplinary approach whereby strategies were used to ensure a range of voices, including Kenyan youth, contributed to all stages of the study process, thus shifting traditional power dynamics in research across countries, populations and individual characteristics. This paper utilizes in-depth interviews (IDIs) which were undertaken with 30 women ages 18–24 years in three Kenyan counties (Nairobi, Mombasa and Migori) to explore decision-making about contraceptive method choice and source choice.

### Sampling and recruitment

The FAFC project works in five focal counties which were selected based on levels of contraceptive use among youth, presence of FP implementing partners and advocacy partners to partner with, regional representation and political commitment to ensuring FP access for youth people. Selection was completed in collaboration with the Kenya Ministry of Health and the National Council for Population and Development. Compared to the two FAFC focal countries (Wajir and West Pokot) not selected for this qualitative study, the selected three counties—Nairobi, Mombasa, and Migori—have higher contraceptive use, an important factor for study eligibility criteria. Nairobi county is the capital of Kenya, is urban, and largest in terms of population at 4.4 million according to the 2019 national census [23]. Mombasa county is situated on the coast of Kenya along the Indian Ocean, is urban, and has a 2019 population of approximately 1.2 million [23]. Migori county is in the western part of the country, is predominantly rural, and borders Lake Victoria and Tanzania. Based on the 2022 Kenya Demographic Health Survey, adolescent pregnancy varies by county, with 8.4% of adolescent girls ever having been pregnant in Nairobi, 10.8% in Mombasa, and 23% in Migori county [26].

Public and private sector SDPs in the three target counties served as the recruitment sites for study participants. Target SDPs were chosen using data from the Kenya Health Information System in order to identify facilities with high client loads. The Ministry of Health’s county reproductive health coordinators provided additional input and support in order to ensure inclusion of facilities and pharmacies which also had high client loads, particularly for adolescent and youth clients. Two to three public facilities, two private facilities [types included private clinics (n = 3), nursing homes (n = 2), and a faith-based dispensary (n = 1)], and one privately owned pharmacy were purposively selected in each county. The type of public facility varied by county and included: two public health centers in Nairobi, a public dispensary and health center in Mombasa, and three public hospitals in Migori. In total, 16 SDPs served as recruitment sites for study participants. Prior to the start of data collection, permission was sought from health facility managers and pharmacy owners.

The recruitment of study participants was stratified by county and parity (no children or 1 + child). The aim was to complete four IDIs among nulliparous women and four IDIs among women with one or more children per county, with the interviews spread across the SDP types within each county. Recruitment was undertaken at different types of SDP in order to identify women who visited various SDP types, but women did not need to be seeking FP services at the time of recruitment.

The eligibility criteria for participation in the in-depth interviews included being a woman between 18 and 24 years, and having ever used at least two modern contraceptive methods that could be obtained from an SDP (implant, IUD, injectable, oral pills, emergency contraception (EC), and male condoms). In this manuscript, we refer to male condoms as ‘condoms’. Of note, the respondents needed not be using a contraceptive method at the time of recruitment. This study included respondents aged 18 and older due to the desire to recruit women with more contraceptive experience.

### Study procedures

A 10-day training of interviewers was held in August 2019 which included classroom-based sessions which covered ethics training, review of study methods, procedures and guides and mock interviews. The eight trained female interviewers were from the three counties and familiar with the local languages and customs. All interviewers had a first degree in social sciences and were experienced in qualitative research with youth on issues related to sexual and reproductive health. We also selected female interviewers under 30 years of age in an attempt to balance power dynamics between interviewers and participants.

A field-based pilot was conducted at two public health facilities in Nairobi which were not part of the facility list for the study. The pilot was undertaken by the trained interviewers in order to pre-test field procedures, including recruitment, screening and interview completion. Over the course of two days, eight IDIs were completed. Based on the successful pilot, the number of questions in the guide was reduced and  one question was moved.

From August to September 2019, one or two interviewers per county rotated across the SDPs. All study participants were recruited outside of, or in the waiting areas of the selected SDPs. Approval to undertake the study at each SDP was sought from the SDP in-charge. The MOH supplied a letter of approval and support for the study which was presented to the SDP in-charge. The eligibility of potential participants was assessed using a short checklist to ascertain information about age, parity and past and present contraceptive use. Once a woman was confirmed to meet the eligibility criteria, was informed about the study and was interested in participating, she was asked by the interviewer to provide verbal consent to participate in the study in accordance with the regulations and requirements set forth by the IRB on record (AMREF Health Africa Ethics and Scientific Review Committee). The informed consent form was read verbatim to potential study subjects. Women were asked if they had any questions, and if so, the interviewer answered all and any questions. If the respondent freely and voluntarily agreed to participate in the study, the interviewer signed the consent form and gave the copy to the respondent. The informed consent process and interviews were conducted in a private setting outside of the recruitment site which frequently included seats in a shaded spot away from where anyone could overhear the conversation. Emphasis was placed on ensuring that respondents’ preferences around privacy and confidentiality were attended to. All interviews were digitally recorded and were conducted in English (n = 1), Kiswahili (n = 18), DhoLuo (n = 8) or Kuria (n = 3) based on the preference of the respondent. On average, interviews took about 75 min.

### Data collection instrument

The guide followed a modified life history approach to understand adolescent girls’ and young women’s family planning use and decision-making processes from first use of contraception until the time of interview [32]. The semi-structured IDI guide included questions about young women’s life circumstances as well as their decision-making processes around selecting contraceptive methods for every method ever used. For each method they adopted, we probed on whether women prioritized their choice of the contraceptive method, which then influenced where they sourced the method; or prioritized their choice of where they would obtain an FP method, and then decided on the type of method to use after going to the source. This last section also asked about features of service delivery points and providers that are attractive and important to adolescents and young women. The IDI guide was translated into Kiswahili, DhoLuo, and Kuria, and pre-tested before data collector training, and finalized after piloting.

### Data analysis

The digitally-recorded IDIs were transcribed and translated from the local languages into English. Transcripts were uploaded into Dedoose software (v.8.3) for coding and analysis. A codebook was developed based on a priori codes which were based on the IDI guide. Five members of the research teams at AFIDEP and UNC reviewed the same two transcripts and coded them based on a priori codes; where needed, they identified and agreed upon additional emergent themes for coding. A third transcript was double-coded by the team members to assess intercoder reliability and to ensure the codes were being applied consistently. The team reviewed and resolved discrepancies in coding and adjusted the codebook as needed. The full codebook was reviewed by a wider group of team members from FAFC in order to ensure consistency in interpretation and understanding between those intimately involved in coding the data and the larger team. The remaining transcripts were divided among team members for coding. The team developed matrices to identify themes, connections, and patterns based on methods used and how young women prioritized choice of contraceptive method versus source, type of source, and relevant characteristics of SDP and providers that influenced decision-making. An independent check of coding was undertaken by a member of the larger team at the end of the coding process.

In the analysis, we defined a decision-making instance as each time a respondent initiated a new contraceptive method. We then examine the circumstances and influences around that decision. We categorize each contraceptive decision-making instance by the decision that was prioritized by the participant: the decision about which method to use or the decision about where to obtain a contraceptive method. In addition, a third category of decision-making arose whereby there was not a clear prioritization of either choice (method or source) and we refer to this group of decisions as “ambiguous decision-making”. An analytic matrix was created to be able to explore differences by characteristics including county, age, parity, education level, and urban/rural. No differences were found by these demographics and so results are presented jointly.

### Ethics approval

All study materials, including guides and consent forms were approved by the AMREF Health Africa Ethics and Scientific Review Committee (P205/2019), National Commission for Science, Technology and Innovation in Kenya, and the University of North Carolina at Chapel Hill Institutional Review Board (19-1360). Additional approvals were obtained from each county’s Director of Health. The in-charge of each individual SDP provided approval for data to be collected at their site.

## Results

### Characteristics of respondents

In total, 30 young women met the eligibility criteria and were interviewed; ten were from Nairobi, nine from Mombasa and 11 from Migori (Table 1). The mean age of participants was 21.6 years (range 18–24 years). About 60% of the sample was married at the time of interview and half of the sample had one or more children. The average age at which the respondents first used a modern method of contraception was 18.2 years (range 13–21 years). Participants were predominately recruited from public sector facilities across the sites (n = 17) followed by private facilities (n = 8) and pharmacies (n = 5). No notable differences were observed when the analysis was undertaken by county, and therefore results are presented by combining respondents from all counties.

**Table 1:** Number of IDI respondents by county and recruitment site

County	Recruitment site/SDP type			Number of IDIs
	Public	Private	Pharmacy	
Nairobi	4	4	2	10
Mombasa	6	2	1	9
Migori	7	2	2	11
Total	17	8	5	30

### Decision-making instances

In this sample of young women, 15 had ever used two methods, 11 had used three methods and four had used four methods. In terms of the number of women who ever used a specific method type, male condoms (n = 22) and injectables (n = 20) were the most frequently reported methods ever used (Table 2).

**Table 2:** Decision-making priorities by method used

Method used	Method choice prioritized	Source choice prioritized	Ambiguous decision-making process	Total number of women who ever used each method
Male condoms	19	0	3	22
Injectable	16	3	1	20
Emergency contraception	15	0	0	15
Implant	11	3	1	15
Daily oral contraception	5	1	1	7
Total number of decision-making instances	66	7	6	

When examining the order in which the young women recollected having made family planning-related decisions, we identified 79 decision-making instances across all 30 respondents (mean = 2.63, median = 2.50 contraceptive use decisions per respondent). The women reported three main categories of decision-making priorities: prioritization of a contraceptive method at the time of seeking a method, which then influenced the SDP selected (n = 66 decisions); prioritization of an SDP, which then influenced the method they selected (n = 7 decisions); and instances where the decision-making process was ambiguous in terms of source vs. method choice (n = 6 decisions) (Table 2). For example, 22 respondents ever used a male condom. At their first use of a male condom, 19 prioritized the method choice, zero prioritized the source, and three had an ambiguous decision-making process. All of the respondents also used other methods, and therefore their information and decision-making prioritization appears in the following rows for other methods. As shown in Table 2, decision-making priorities were similar for the other methods.

Below we describe the contraceptive methods used by these three decision-making priorities. In the first section about young women who prioritize choice of method, we present the findings by method and draw out themes regarding what influenced prioritization of the method chosen and themes about subsequent source choice. Not surprisingly, those who first used condoms shared a different set of circumstances and needs that led them to prioritize method choice compared to those who first used a hormonal method. In the section about prioritization of SDP choice, which was less frequent, women were typically at the SDP for another reason (e.g. well baby check or side effects) and this led to the SDP being prioritized. Finally, we present those who had an ambiguous decision-making process and the range of themes that arose in that category.

#### Prioritizing choice of contraceptive method

Among the study participants, all of whom had used two or more methods, most instances of seeking FP were driven by the decision to use a particular method which then influenced their choice of source. Most reported being unequivocal about their choice of method; this was especially true for the first contraceptive method used (n = 24 women), which was most often male condoms (n = 18 women) (data not shown). Among the 30 respondents in this study, 20 respondents always prioritized method choice for all of the methods they ever used (range from 2–4 methods) and 29 respondents prioritized choice of method for at least one of the methods they ever used. Notably, the choice of the first method used among these young women are often related to numerous factors including their age, marital status, partner engagement in decision-making and obtaining the method. More details of the influences on the specific method chosen over the life course are presented elsewhere [31]. Yet when male condoms, EC and implants were the methods selected, features of the method often drove the method choice and therefore prioritization of the method over the source. Below we describe in more detail how these method choices were formed and how the source choice followed. The results are presented using the same order as the methods presented in Table 2.

##### Male condoms

At the time of first contraceptive method choice, respondents reported that they had limited knowledge of other types of contraceptive methods and learned about them from school, partners and friends. These young women, who were often unmarried then, also identified that male condoms were appropriate for them because they were worried about sexually transmitted infections, including HIV, and male condoms provided dual protection. When asked about how she decided to use her first contraceptive method, one young woman from Nairobi explained the following:

*One, I didn’t know about the other types of emergency [contraception] and injectable and the rest. So I decided let’s do a condom because I hear it’s safer, you can’t get pregnant. That was the first worry. The second worry was this person, I am not so sure whether he is HIV positive or not. By the way you have doubts. You have to give that benefit of doubt.*—23-year-old with no children in Nairobi

Since the male condom was the only available method that provides dual protection, these women decided to use male condoms. Once the method choice decision was made, these young women (or their partners) mostly sourced male condoms from drug shops or pharmacies that were geographically close to where they lived or attended school. When the young women were asked why they chose to source male condoms from drug shops or pharmacies instead of hospitals or clinics, they often responded that drug shops were “easy” and hospitals were “difficult”. One woman in Migori stated “*You know someone cannot just go to the hospital for a condom, and they [male condoms] are available nearby in a chemist [pharmacy]*”. This speaks to how the ease of accessing male condoms from drug shops and pharmacies was a critical factor when women and their partners decided where to procure male condoms.

##### Injectables

Most women who used injectables at some point in their contraceptive history reported that they identified injectables as the method they were ready to use, which then influenced their decision on where to source it (n = 16). Many of these women shared that they learned about injectables as an option for contraception when they were counseled on FP methods during their antenatal care visits (n = 7), or from friends who had children (n = 3). Many of these women chose to use the injectable in the post-partum period because they wanted a longer-acting method, compared to male condoms or oral pills, to space their next pregnancy, and a few reported even knowing the exact duration of protection they were seeking. A 22-year-old woman from Migori described her decision-making process for choosing injectables, which she started approximately 1.5 months after giving birth:

*There is a day women were being put on various family planning methods. I went to the hospital and told nurses to put on the injection that lasts for a period of three months. The nurses agreed and put me on it.*—22-year-old with 3 children in Migori

Another woman from Mombasa who used the injectable as her first FP method after having her first child, explained how she jointly decided with her husband to use the injectable before arriving at a private hospital to source it:

*Interviewer (I): Did you talk to anyone when you were deciding to use [injectables]?*

*Respondent (R): Yes.*

*I: Who did you talk to?*

*R: My husband.*

*I: What did you talk about.*

*R: About which method we can use to plan our family, and that is when we decided to use the three-month injection.*

*I: Did you decide from home or you came to the hospital?*

*R: I decided at home and when I came to the hospital, I had already made my decision on the injection.*—23-year-old with 2 children in Mombasa

Most women reported having sourced injectables from hospitals (n = 12), with a few sourcing them from dispensaries or pharmacies. Women provided a plethora of reasons why they chose these FP sources, including the close distance of the facility to their home, having previously received their FP method (whether daily pills or implants) from the same facility, receiving other health services including antenatal and post-partum care at the same facility, and positive interaction with and trust in health care staff.

##### Emergency contraception (EC)

As expected, EC was the method of choice for young women who had unprotected sex. Women and their partners realized that there was the potential for a pregnancy and that their best option at that point to avoid an unintended pregnancy was to take EC, meaning that the decision regarding which method to use took priority over the decision of where to source it. When describing why EC was the first method selected, a 21-year-old woman from Migori replied that, “*It was only that it could help one not to get pregnant without [a] plan*”.

Whether EC was used among new FP adopters or experienced FP users, women or their partners sourced EC from pharmacies. Most women shared that the main reason they chose a pharmacy was because it was proximate to their home (n = 9). A couple of women reported that the pharmacy was the only source they were aware of that sold EC (n = 2), and two different women shared that they preferred to source EC from pharmacies instead of hospitals because the health care staff at pharmacies would not be judgmental in the way they envisioned nurses and doctors at hospitals to be. One woman from Nairobi reported:

*Because when I go to the hospital, the doctors…I don’t like how they talk to these girls. You know they should understand us. So when I go there, they will be like ‘Mmmh, what have you started doing this early?’ So I didn’t want that. I want somewhere where it’s business. So let me give you money, you give me what I want.*—23-year-old with no children in Nairobi

##### Implants

Only experienced FP users chose implants, and most decided on this particular method before determining where they would obtain it. Like injectable users, most of these women wanted a long-acting FP method to space their next child. A 22-year-old woman from Migori described making the decision to use the three-year implant jointly with her husband:

*R: While I was pregnant, he [her husband] used to tell me that after I had given birth, he’ll introduce me to a family planning which lasts... I told him five years is a longer duration and I should take the one that last for three years.*

*I: Mmh*

*R: He accepted, immediately after giving birth, he invited a nurse and put me on the family planning method.*—22-year-old with 3 children in Migori

All implant users chose to source their implant from public or private hospitals which were close to their homes. A number of these women had received antenatal, childbirth and/or child health care from the same hospitals (n = 9).

##### Oral contraceptives (OC)

Five out of seven young women who ever used oral contraceptives (OC) reported that they knew they wanted this method and then sought out a source of the method. Some women chose to use OC because they were perceived to have fewer side effects than other methods (n = 3), and one woman used OC because her prior method failed. These women sourced pills from pharmacists, some stating that they went to the same pharmacies from where they bought other medicine. Other women sourced OC from hospitals because they were the only places the women knew of to get OC. One woman from Nairobi described knowing she would obtain OC from a pharmacy:

*I: So when you left the house that day, when you were going to get the family planning method, what had you planned to do when you left the house? To take…*

*R: [Interjection] I left the house to go buy those pills.*

*I: You left knowing you were going to buy pills?*

*R: Yes. I told myself to try those ones and I see how I will feel.*

*I: So you had already decided before you got to the chemist [pharmacy]?*

*R: Yes.*—24-year-old with no children in Nairobi

#### Prioritizing choice of contraceptive source

In total, six women representing seven decision-making instances prioritized the source from which they got FP for at least one method they had previously used, and the decision about which specific contraceptive method to use was secondary. Generally, the respondents in this category are women seeking counseling and contraception, either to initiate a new method or to switch methods. Women who prioritized the choice of FP source generally fell into two sub-categories: women with newborns or infants, and women learning about or experiencing side effects from the contraceptive method they were using at the time. One woman occupied both sub-categories because she prioritized the source of FP over the specific method for two different methods used at different times of her life. Four women had recently given birth and went to the hospital to seek advice on which method to get. They often returned to the facility where they had given birth and/or were going for post-partum and newborn check-ups.

One of these women from Migori was a first-time user of FP. This woman had received antenatal care from a nearby hospital where she learned about different types of methods. This public hospital was her facility of choice, she explained, because it was not far away, it adhered to standard guidelines, and offered better quality services. At the time she decided she wanted to use FP, she still had not chosen the specific method but returned to the public hospital where she had previously received care. She reported:

*R: As usual I just left for the clinic, so upon reaching the clinic I took the injection. Even my husband was not aware. So in my mind it was already clear that upon giving birth to my second child, I will take a family planning but I had not yet decided on which method.*

*I: So at what point did you decide on the method, was it at the point you had a talk with the doctor or?*

*R: Yes at the time I had a talk with the doctor during the clinic day. So the doctor asked me whether I want an implant or an injection. So I asked him of the timeframe for the injection and he informed me of the 3 months injection method and also the three or the five year implant method. I simply told him to give me an injection.*—21-year-old with 3 children in Migori

Another young woman from Mombasa, who had initially used male condoms as her first method, also described how learning about the provision of free FP at the facility where she had recently given birth influenced her decision to get long-term contraception there:

*When I just gave birth it was announced to us at the hospital that you can [get] family planning free of charge…I talked with my husband then I came and [the implant] was inserted.*—20-year-old with 2 children in Mombasa

When asked why she decided to have her implant inserted at that particular public hospital, the young woman explained that it was the same hospital from which she had received antenatal and childbirth care, and to which she brought her children for check-ups. While there were other hospitals nearby, the woman explained that she chose this hospital because “now this is the hospital I am used to.”

Three women in this group had heard of or experienced side effects from the contraceptives they were using and wanted to switch to a different type of method or otherwise get contraceptive counseling. At times, these women went back to the source where they had procured the contraceptive method they were using at the time, and then switched to another method. One of these women from Migori described her experience of also being switched from pills to injectables:

*I asked [the provider] about the side effects of the pills and he told me that when I first take it, I will bleed for the first two to three months after which it will stop. Mine didn't stop. It was 24 hours. So I went back to him and he told me to continue using it but to monitor. When I went back to him I had lost so much weight. He told me, ‘Don't force yourself to continue with this one. Let's try this three months injection and see if it will be better for you. They are usually like this and this one has not worked well with you.’*—24-year-old with two children in Migori

In this case the woman had previously decided to use the daily pill and then went to a pharmacist that was very close to her home to source the method. Later, she sought advice from the public hospital where she had delivered her baby to address the side effects she was experiencing from the daily pill.

Notably, the respondents who switched from the daily pill to injectables did not initially make the decision to use injectables. They first chose their SDPs/provider, and then after telling their providers about side effects they were experiencing, the providers advised them about which contraceptive method the women should try next.

#### Ambiguous decision-making process

Six women in six separate decision-making instances described an ambiguous decision-making process where they did not make an explicit initial decision about a specific method or source before using a contraceptive method. These varied from situations where the women had no agency, to ones where women went through a consultative and multi-stepped process. Among the six women who described an ambiguous decision-making process for at least one of the methods they used, this decision was for the first method ever used for five respondents.

Two women described situations where they lacked the ability to make an active and independent choice about use of contraception: one young woman was sexually assaulted and asked the perpetrator to wear a male condom, and the other woman was told that the injectable contraceptive she was receiving while at school was a tetanus shot.

One young woman described a case where she had the capacity to make her own decisions but lacked information to support decision-making. In this case, the young woman accompanied her friend for family planning and then, based on the advice of the provider, decided to get the method for herself:

*I used implants because there is a friend of mine who asked me to accompany her to the hospital. So, I accompanied her to the hospital and had the implant placed for her. So, after she had it placed, the doctor advised me to place it also and I did it.*—20-year-old with no children in Nairobi

Another woman described deciding to use male condoms as her first method after receiving them for free, along with counseling, from community health workers in her neighborhood.

Finally, one young woman from Mombasa explained that she and her husband were deciding between OC and injectables after the birth of their child. While they discussed the advantages and disadvantages of each method at home, they did not make a final decision until they were counseled by a service provider:

*R: [My husband and I] talked in the house and then we went to the hospital and talked to the doctor.*

*I: And where did you make the decision to use the pills as a method of family planning?*

*R: Hospital*

*I: Hospital? You were with a doctor when making the decision?*

*R: Yes*

*I: What did the doctor explain to you?*

*R: He explained to me about family planning and told me there are various methods if I accepted to practice family planning. I asked him which method was available or which method could use. He explained to me about the pills telling me I have to follow the prescription and I decided to use the pills.—*24-year-old with 3 children in Mombasa

## Discussion

This qualitative study utilized in-depth interviews with young women from three counties in Kenya who were experienced FP users to understand the prioritization and decision-making process for method selection and contraceptive source selection. Our study found that the vast majority of young women knew what contraceptive method they wanted to use and then subsequently made the decision of where to obtain it; this was true both for the first method ever used and subsequent contraceptive decisions. Of the smaller number of women who selected the source of their contraceptive method first, many were in the post-partum period or experiencing side effects so turned to a trusted health provider for contraceptive counseling.

Our findings are consistent with previous quantitative studies that show that women often have a method in mind when seeking family planning services [21]. By utilizing qualitative data, our study was able to go beyond common quantitative questions and understand what factors influence method selection and explore why that choice was generally prioritized over source selection. Respondents were often unwavering and confident in their method choice, having made the decision based on receipt of information through a variety of sources including friends, partners, providers, and schools, as shown in earlier studies [3–5, 9]. They then determined where to obtain the chosen method. This was particularly true for the first methods women used, most often male condoms and EC, where respondents cited limited knowledge of other contraceptive methods, dual protection and protection against pregnancy after unprotected sex [33]. The confidence women exhibited regarding method selection for male condoms and EC also extended to other commonly used hormonal methods in Kenya, such as implants and injectables, where, similar to findings from other studies, women often expressed a desire for a method that was longer acting and did not require frequent follow-up at health facilities [7, 9]. Our study expands on the Jarvis et al. study, which found that women frequently know what method they want before seeking services, by suggesting that the choice of a method may guide where it is sourced [22]. Our study found that when considering which decision was prioritized, the default for women who decided to use contraception was to seek services after identifying the method they wanted; only in specific circumstances did women arrive at a SDP without having pre-determined their method of choice.

We found that for a smaller set of young women, after deciding to use contraception, the choice of a method was secondary to the decision about where to obtain the method. In most cases, this prioritization was applicable to young women who were farther along in their reproductive life course and had either recently had a pregnancy or birth or were experiencing side effects and required information and counseling from a provider in order to determine what contraceptive method to use. When explaining this prioritization, women cited their inability to decide on a method before seeking services, and instead prioritized returning to trusted health care staff or sources where they had received services in the past, primarily for some type of maternal, newborn or child health service.

Our study also highlighted that counseling during pregnancy and after childbirth is key in helping women make decisions about what method to use and where to seek contraception, regardless of which decision they prioritize. This includes women who selected their method before selecting the source but after receiving information about contraception during past antenatal or post-partum care visits. Despite most women having decided on a contraceptive method prior to visiting the facility, our study highlights the importance of provision of high-quality information and services, including ensuring comprehensive counseling on a full range of methods when a woman comes to a facility to seek antenatal, postnatal, child health visits, and contraceptive services [34], as it may influence future contraceptive decision-making. Additionally, women who return to the SDP where they gave birth for FP counseling and contraception benefit from these sites offering a broader range of methods as compared to pharmacies, and therefore these sites have the ability to provide them with their chosen method.

Additionally, despite few examples of ambiguous decision-making around method or source prioritization, the examples highlight the value and strength of qualitative data to uncover and explore the nuances of contraceptive decision-making. At least two of the experiences that young women shared highlight issues around power and abuse, whereby these young women were not able to make a free and informed choice about contraceptive use. These examples point to complexities around contraceptive use and decision-making among young women, which are important for health care providers to be sensitive to.

Regardless of which decision—the method or the source—was prioritized, many of the desired features of service delivery points were similar for the source where women ultimately received their method. As found in other studies, young women preferred sources that were proximate to their home and were perceived to offer privacy and confidentiality [7, 9, 13]. As women transitioned to having children or being married, new preferred features emerged such as a facility with which they were familiar and where they could receive all of their care or that they had received FP information from the facility in the past.

This study has several strengths and limitations. First, in using in-depth interviews we were able to ask complex questions that went beyond common quantitative questions in order to understand the factors that influence prioritization of contraceptive decision-making and sourcing, and how these two choices were related. To the best of our knowledge, this is the first study to address this research topic particularly with the unique population of adolescents and youth. Additionally, our study included FP users who were using clinical methods as well as those who had received non-clinical methods from pharmacies and shops, a segment of the population that is often missed through client exit interviews. Finally, by asking participants to discuss all contraceptive methods they had ever used, we were able to explore multiple decisions about method and source made at different points in a young woman’s life.

Despite the strengths of our study, collection of retrospective data introduces recall bias. Respondents may have had challenges recalling important details about their past decisions. Given that this research topic focuses on nuances in decision-making, it is possible that some respondents may not have been able to accurately recall how and why they made particular decisions. Further, knowledge gained and decisions made later in their lives may have biased their recall of earlier decisions. In addition, the study population of focus is young women who had previously used two or more methods of contraception, including clinical methods. These “experienced” users may be different than women who have not used multiple methods.

Finally, for a small number of respondents, the reason for prioritizing one choice over the other was ambiguous and even with probing, respondents often had difficulty differentiating which decision was prioritized. Respondents typically resorted to explanations about the features of the methods or the source that they preferred or liked, but sometimes did not clearly articulate why they would prioritize one decision over the other. Additionally, male condoms are often procured by male partners and are easily accessible at multiple sources, thus making the decision-making priority less relevant [9, 33]. Relatedly, some respondents indicated that decisions regarding method and source appeared to occur jointly. It may be that women have an innate association in their minds, for example, that certain methods are obtained from certain sources, such as male condoms from pharmacies [35]. They may also attribute specific features of sources to a particular type of source, such as that methods are free at public facilities or that quality of care is higher at private facilities, and these characteristics may be closely related to method selection.

## Conclusion

Our findings highlight that the choice of method among women at the beginning of their contraceptive life course is primarily limited to male condoms, being the main method they know about. Programs should develop targeted information campaigns either through the mass media, through community-based programming, or in schools for adolescents and youth that focus on increasing knowledge about contraceptive options, including information on the features of methods, the importance of male condoms for dual protection, side effects of different methods, while also dispelling myths and misconceptions about methods and method use among young people. Broadening young people’s knowledge base on contraceptive methods and where to source them will ensure that they are able to select the method that best suits their life circumstances, even if in the future.

This study also points to the need to provide young women with information about contraceptive options at all points along the reproductive health continuum of care. Several respondents in our study made decisions about which type of contraceptive to use based on family planning counseling during antenatal care, childbirth care, postnatal care or a well child visit. Where possible, programs should include comprehensive counseling on family planning during each of these contacts with the health system and consider other opportunities for integration, with a particular focus on reaching sexually active young adults with information on the range of family planning methods available as it may inform future contraceptive decision-making.

With the recent roll-out of the 2018 policy now allowing pharmacies to sell and administer injectables [29], a commonly used method that has been primarily and historically sourced from public facilities in Kenya [23], future work should explore how and if this broadens method mix among the youngest clients as well as if this shifts the decision-making process regarding source and method prioritization. Expanding source options for hormonal methods may help to address some of the barriers that women face seeking contraception, particularly young women.

## Data Availability

Information about the study, survey tools and data are available at: https://dataverse.unc.edu/dataverse/fafc. A formal request needs to be made and a data sharing agreement will have to be made before sharing the data.

## Abbreviations

AFIDEP: African Institute for Development Policy
EC: Emergency contraception
FP: Family planning
IDI: In-depth interview
I: Interviewer
OC: Oral contraception
IUD: Intrauterine device
R: Respondent
SDP: Service delivery point
UNC: University of North Carolina at Chapel Hill
